# Reversible iodine capture by nonporous adaptive hybrid[3]arene crystals

**DOI:** 10.1039/d6sc00236f

**Published:** 2026-04-14

**Authors:** Ruike Zhang, Wenqian Liu, Jiong Zhou

**Affiliations:** a Department of Chemistry, College of Sciences, Northeastern University Shenyang 110819 China zhoujiong@mail.neu.edu.cn

## Abstract

Iodine capture is of great importance for environmental protection and chemical safety, motivating the development of efficient materials for iodine adsorption. Herein, we report an adsorption strategy that uses nonporous adaptive crystals based on novel hybrid[3]arene 1 (1α). 1α exhibits the ability to effectively capture iodine from both iodine vapor and iodine dissolved in *n*-hexane, reaching maximum capacities of 1.87 g g^−1^ and 0.74 g g^−1^, respectively. The adsorption of iodine molecules by 1α is driven by C–H⋯I and O–H⋯I interactions. Notably, 1α can be effectively recycled at least 10 times without a significant reduction of iodine capture capacity. It is anticipated that this work will provide valuable insights into the design of nonporous adaptive crystals as versatile platforms for radioactive iodine capture with recyclability.

## Abstract 2

A novel nonporous adaptive crystal based on hybrid[3]arene can efficiently capture iodine from iodine vapor and *n*-hexane, demonstrating good iodine capture capacity and recyclability.

## Graphical abstract

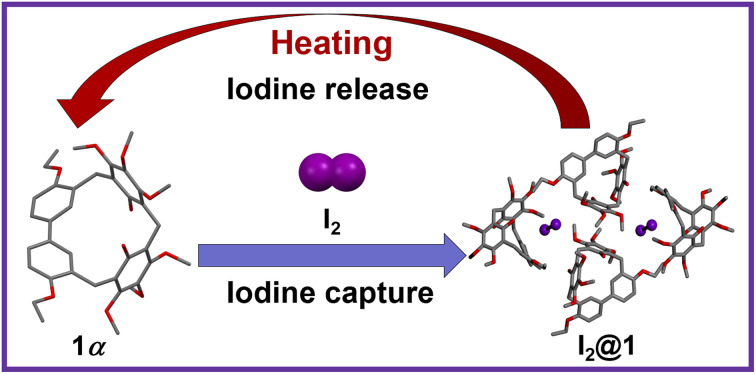

## Introduction

Radioactive iodine, a byproduct of the nuclear fission process, poses a weighty and long-lasting threat to the environment and human health due to its extended half-life and volatile nature.^1,2^ The potential release of radioactive iodine can occur during nuclear incidents or leakages, and once released, this hazardous substance can readily permeate and accumulate within food chains, ultimately endangering human life.^3–9^ While nuclear energy is often regarded as a sustainable and relatively eco-friendly resource, particularly in the context of mitigating global warming caused by excessive fossil fuel consumption, the associated risks of nuclear leakage cannot be overlooked.^10–13^ Given the severe and enduring consequences of radioactive iodine contamination, there is an urgent necessity to advance the development of innovative materials that can efficiently capture and securely store this radioactive element.^14,15^

Nowadays, considerable research efforts have been directed towards developing advanced materials capable of efficiently capturing and storing iodine in various forms.^16–19^ Among them, porous materials including metal–organic frameworks,^20–22^ covalent organic frameworks,^23–25^ porous polymer networks,^26,27^ and hyper-cross-linked polymers are used for iodine capture applications.^28^ These materials exhibit high specific surface areas and tunable pore environments, which facilitate selective molecular recognition and exhibit strong adsorption capacities.^29^ However, their practical applications are limited by adsorption reliability and long-term stability.^30,31^

To overcome the problems in practical applications, the development of stable and efficient adsorbents is essential. Previous studies have shown that dense organic crystals can undergo guest-induced structural adjustments despite lacking permanent porosity, indicating that lattice adaptability is an intrinsic feature of certain organic solids.^32^ Building on these foundational observations, recent efforts have sought to systematize and extend this behaviour into what are now described as nonporous adaptive crystals (NACs), which provide a versatile platform for selective molecular capture.^33,34^ These crystals initially possess no intrinsic pores, but are capable of undergoing structural transformations in response to guest molecules.^35–39^ Consequently, NACs can dynamically form adaptable voids or channels that selectively accommodate specific guest molecules based on their sizes, shapes, and chemical characteristics.^40–43^ This dynamic structural adaptability, facilitated by supramolecular host–guest interactions, renders NACs promising materials for molecular capture applications.^44–52^ Notably, by harnessing non-covalent interactions between iodine molecules and polar groups, these crystals can spontaneously form optimized binding sites for efficient iodine capture.^53–55^

Herein, we designed and synthesized a novel hybrid[3]arene 1, characterized by a combination of one 4,4′-biphenol diethyl ether unit and two 3,4,5-trimethoxyphenol units, connected through methylene groups. Based on this unique structure of 1, we constructed NACs of hybrid[3]arene (1α), which demonstrated iodine capture capability. 1α effectively captured both iodine vapor and iodine dissolved in *n*-hexane. Structural analysis revealed that 1α underwent adaptive reorganization into an orderly structure, which was stabilized by C–H⋯I and O–H⋯I interactions upon iodine capture. Remarkably, 1α maintained the iodine capture efficiency for at least 10 times without significant performance loss.

## Results and discussion

**Fig. 1:**
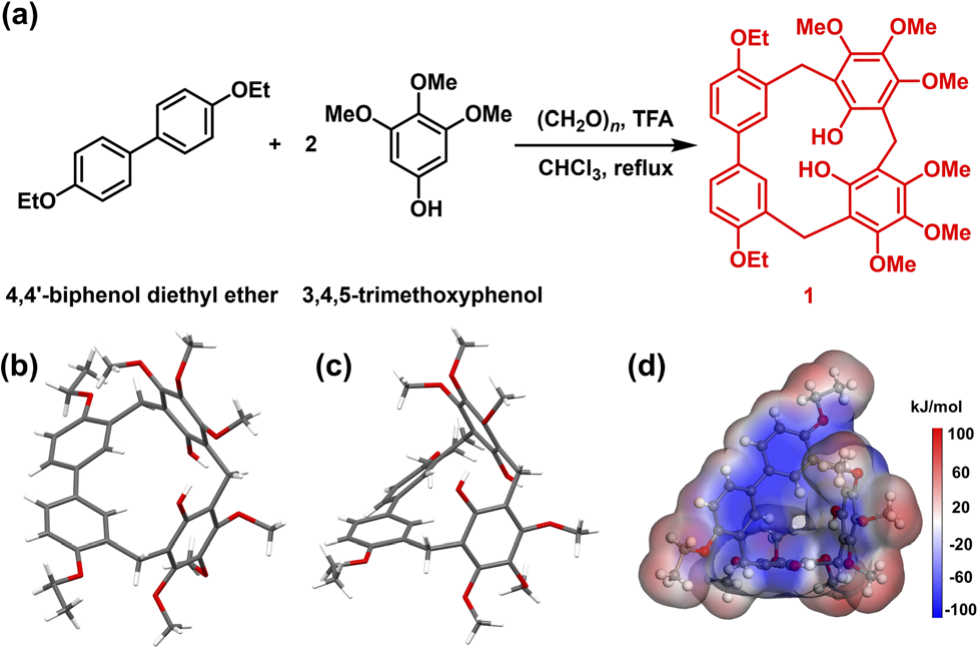
(a) Synthetic method of hybrid[3]arene (1). Crystal structure of 1 on (b) top view and (c) side view. (d) Electrostatic potential map of 1.

Generally, 1 was synthesized by 4,4′-biphenol diethyl ether (1.0 eq.), 3,4,5-trimethoxyphenol (2.0 eq.), and paraformaldehyde (3.0 eq.) in chloroform, catalyzed by trifluoroacetic acid (TFA, 10.0 eq.). The reaction mixture was refluxed at 65 °C for 50 min, generating 1 as a white solid with a yield of 60% (Fig. 1a). The chemical constitution of 1 was characterized by ^1^H NMR, ^13^C NMR, high-resolution mass spectrometry and single crystal X-ray diffraction (SCXRD) analysis (Fig. S1–S3 and Table S1). As shown in Fig. 1b and c, the 1 molecule consisted of one 4,4′-biphenol diethyl ether unit and two 3,4,5-trimethoxyphenol units. These units were bridged by methylene linkers, resulting in a twisted trigonal prismatic structure. Specifically, the biphenyl moiety within the 4,4′-biphenol diethyl ether unit exhibited a twisted conformation, where the two phenyl rings were rotated at a dihedral angle of 40.7° (Fig. S4). Furthermore, the two 3,4,5-trimethoxyphenol units were mutually inclined and located in distinct planes.

The hydroxyl substituents on these phenolic rings were tilted and displaced from the aromatic plane. The electrostatic potential of 1 was partitioned into negative charge regions on the benzene rings and positive charge regions on the methoxy and ethoxy functional groups (Fig. 1d). Electron-rich benzene rings can enhance iodine adsorption by interacting with the localized positive electrostatic potential regions of iodine molecules. Prior to the experiment of iodine capture, 1 was dried under vacuum at 150 °C for 4 h to get activated crystalline 1 (1α). Thermogravimetric analysis (TGA, Fig. S5) verified that the solvent was removed. The powder X-ray diffraction (PXRD, Fig. S6) and N_2_ absorption–desorption curve showed that 1α was a nonporous crystal, with a Brunauer–Emmett–Teller surface area of 1.03 m^2^ g^−1^ (Fig. S7).

**Fig. 2:**
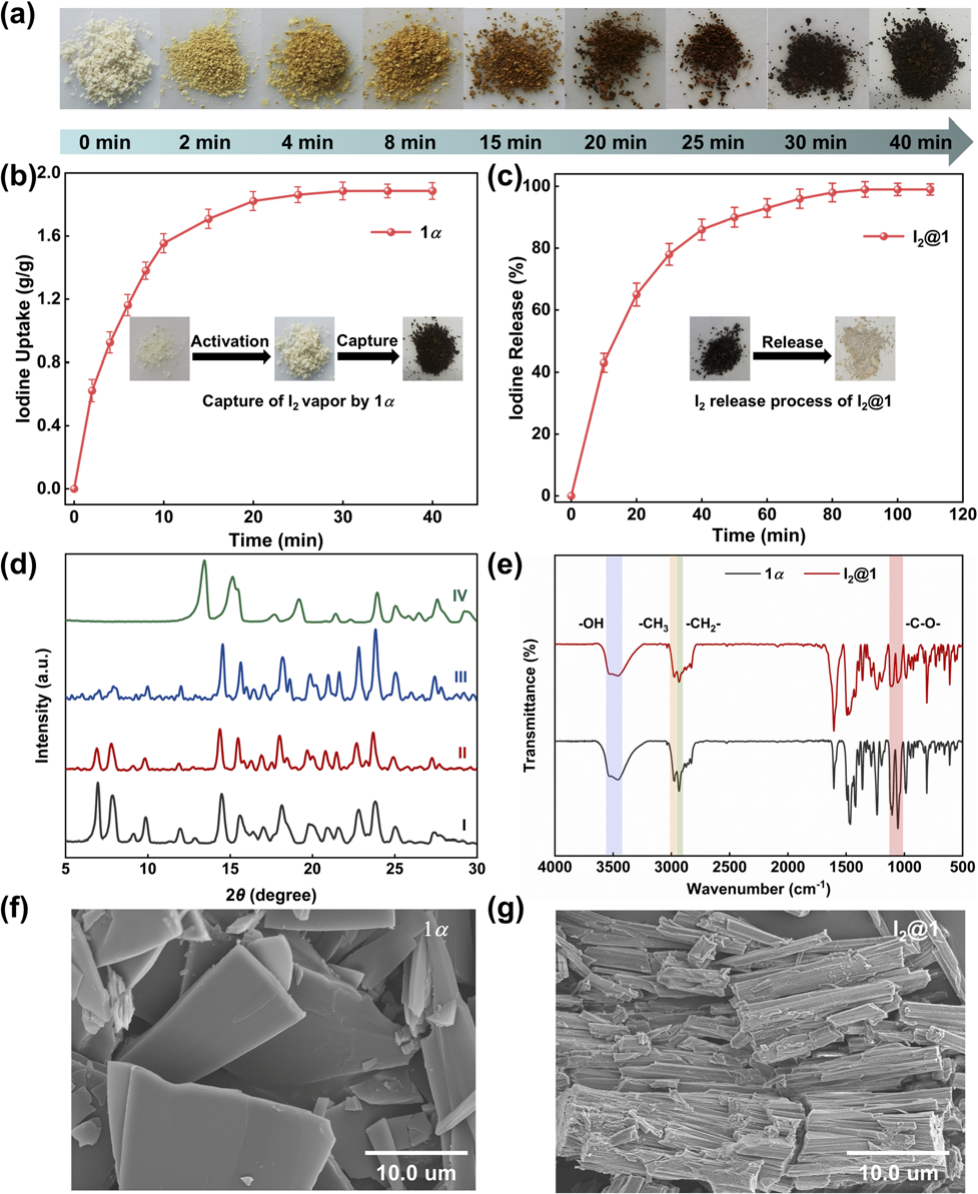
(a) The color change of 1α during the iodine capture process. (b) The iodine capture performance of 1α at 77 °C and 1 bar, inset shows photos of 1, activated 1 (1α), and iodine-loaded 1 (I_2_@1). (c) The iodine release performance of I_2_@1 at 125 °C, inset shows photos of I_2_@1 and after iodine release. (d) Time-dependent powder X-ray diffraction patterns of 1α: (I) 1α; after adsorption of iodine vapor for (II) 10 min, (III) 20 min and (IV) 40 min. (e) FT-IR spectra of 1α and I_2_@1. SEM images of (f) 1α and (g) I_2_@1.

To evaluate the adsorption effect of the newly synthesized 1α on iodine, the iodine vapor capture experiment was conducted at 1 bar. As depicted in Fig. 2a, when 1α was exposed to iodine vapor at 77 °C, the color of the solid progressively darkened over time. As illustrated in Fig. 2b, the iodine uptake rate of 1α increased nearly linearly during the first 10 min, and then reached the adsorption saturation state at about 40 min with a maximum iodine uptake capacity of 1.87 g g^−1^. Although the iodine uptake capacity of 1α was comparable to that of many nonporous solids, the adsorption rate of 1α was faster than that of many previously reported nonporous materials (Table S3). Likewise, the iodine release experiment was investigated at 125 °C, and almost all the iodine was removed at 110 min (Fig. 2c). The time-dependent PXRD patterns of 1α changed over time, but remained unchanged after 10 min (Fig. 2d, S6, and S8), reflecting structural adaptation of 1α to guest uptake.

To elucidate the changes in the crystal morphology and elemental distribution of 1α after iodine capture, analyses including scanning electron microscopy (SEM), energy-dispersive X-ray spectroscopy (EDS) mapping, and Fourier transform infrared spectroscopy (FT-IR) were undertaken. SEM images showed that 1α exhibited a regular block-like particle morphology with smooth surfaces (Fig. 2f). Notably, after iodine loading, the microstructure of the iodine-loaded 1α (I_2_@1) displayed an elongated rod-like stacking arrangement (Fig. 2g), which may provide more accessible sites for iodine adsorption, thereby contributing to the high capture capacity. EDS elemental mapping confirmed that iodine was uniformly distributed in I_2_@1 (Fig. S9–S11). The FT-IR spectra of 1α and I_2_@1 showed only slight variations upon iodine loading. The peaks located near 3470 cm^−1^ and 2990–2998 cm^−1^, assigned to O–H and C–H stretching vibrations, respectively, remained basically unchanged, while the band at ∼1100 cm^−1^ showed a small decrease in intensity after iodine uptake, but recovered to a certain extent after release (Fig. 2e and S19). The thermal stability and iodine adsorption capacity of 1α were evaluated by TGA. Obviously, 1α exhibited no significant weight loss below 300 °C, confirming its high thermal stability. However, the weight loss of I_2_@1 in the range of 25–220 °C was about 66 wt%, corresponding to an uptake of approximately 4.9 mol/1α (Fig. S12). Additionally, the Raman spectrum of I_2_@1 in the low-frequency region (40–200 cm^−1^) showed a peak at 167 cm^−1^ (Fig. S13), which corresponded to the stretching vibration of iodine molecules.

**Fig. 3:**
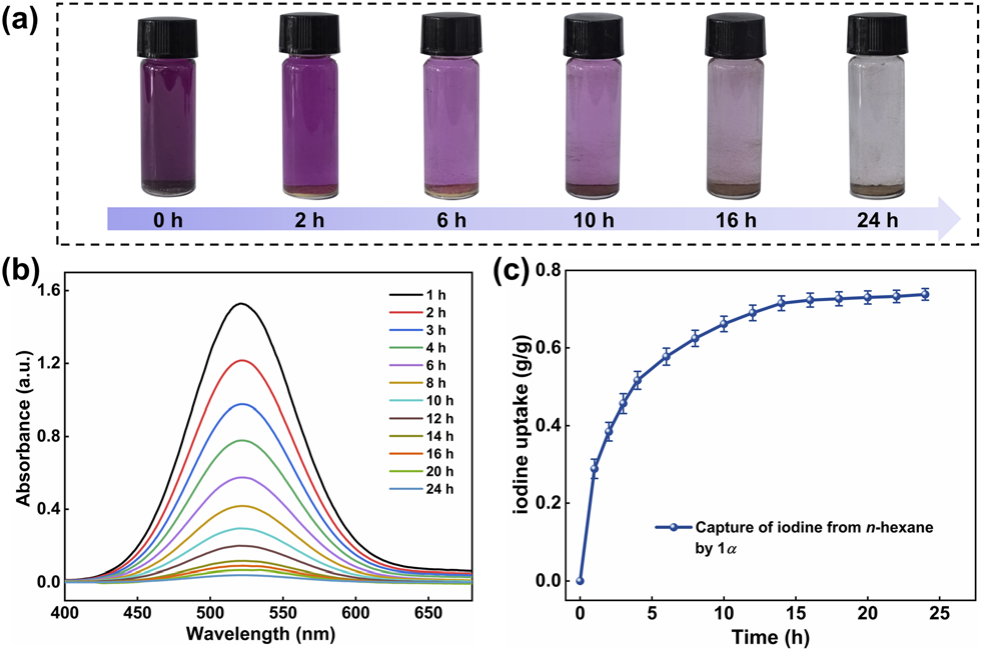
(a) The photos show the color change of iodine adsorption by 1α in *n*-hexane. (b) The time-dependent UV-vis spectra of iodine in *n*-hexane during the capture experiment. (c) The iodine captures capacity of 1α in *n*-hexane at different time points.

Given the excellent solubility of iodine in most organic solvents, the effective capture of iodine from solvents is also important.^56–59^ Therefore, we investigated the iodine capture performance of 1α in *n*-hexane. At 25 °C, 5 mg of 1α was added into the solution of iodine in *n*-hexane (5 mmol L^−1^, 3 mL). As shown in Fig. 3a, the color of 1α changed from white to yellow within 24 h, while the color of the solution transitioned from deep purple to transparent. The UV-vis spectra of the solution over the adsorption period were exhibited in Fig. 3b. After 24 h, the uptake of iodine from *n*-hexane by 1α reached saturation, with a maximum capacity of 0.74 g g^−1^ (Fig. 3c). What's more, 1α could be easily recycled after iodine capture. When I_2_@1 was immersed in methanol, the iodine was rapidly released and the solution turned brown within 2 h (Fig. S16).

**Fig. 4:**
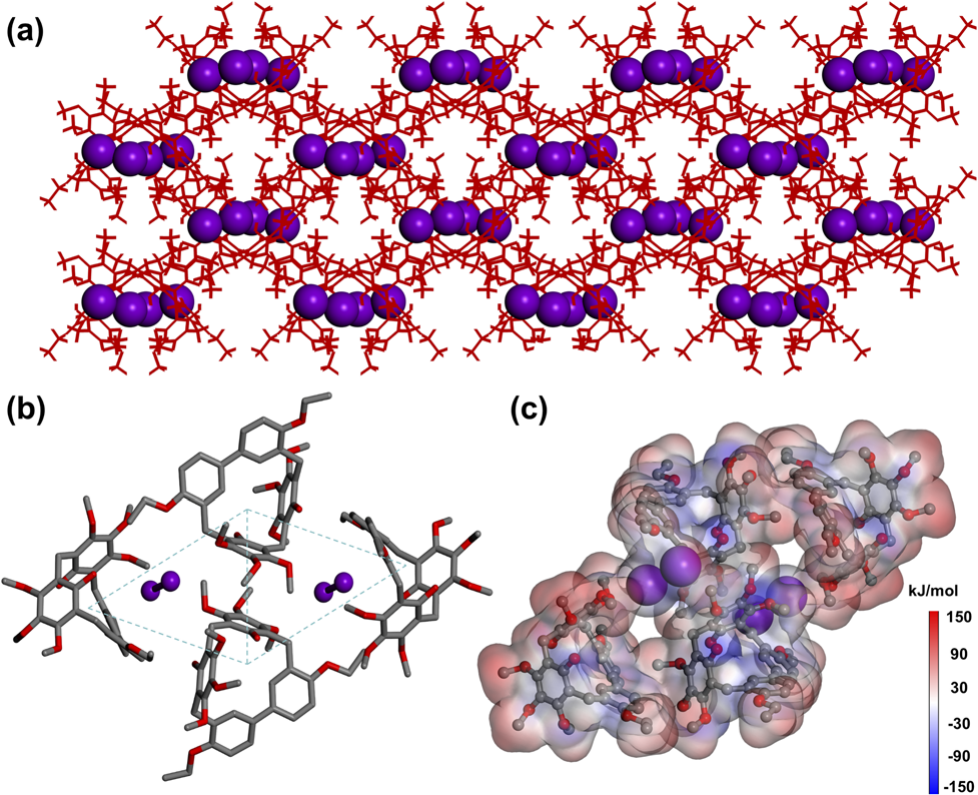
(a) Crystal packing view of I_2_@1 shows the arrangement of 1 and I_2_ molecules. (b) Illustration of the I_2_ capture mode in 1 shows the position of I_2_ molecules in 1. (c) Electrostatic potential mapping of I_2_@1.

To elucidate the structural features after guest uptake, single crystals of iodine-loaded 1 (I_2_@1) were obtained by slowly evaporating a chloroform solution containing 1 and iodine. In I_2_@1, each 1 molecule adopted a slightly distorted geometry upon I_2_ capture. These molecules aligned cooperatively to form continuous channels within the crystal lattice (Fig. 4a). Fig. 4b illustrated the interaction between I_2_ and 1. The iodine molecule was not located in the cavity of 1 molecule, but was situated among three adjacent 1 molecules, forming a host–guest complex with a ratio of 2 : 1. Furthermore, in the structure of I_2_@1, four 1 molecules adopted a centrosymmetric parallelogram arrangement, thereby creating cavities capable of accommodating I_2_ molecules. The main driving force for the formation of the host–guest complex after iodine capture came from the C–H⋯I and O–H⋯I interactions between 1 molecule and I_2_ molecule (C–H⋯I distances: 2.373 Å, 2.841 Å; O⋯I distances: 3.137 Å, 3.295 Å, 3.479 Å; Fig. S15). To further analyze these interactions, Hirshfeld surface analysis was performed for I_2_@1, which showed C–H⋯I and O–H⋯I interactions (Fig. S15). The electrostatic potential map of I_2_@1 revealed that the iodine molecule tended to be attracted by the oxygen atoms of the ethoxy groups (Fig. 4c).

**Fig. 5:**
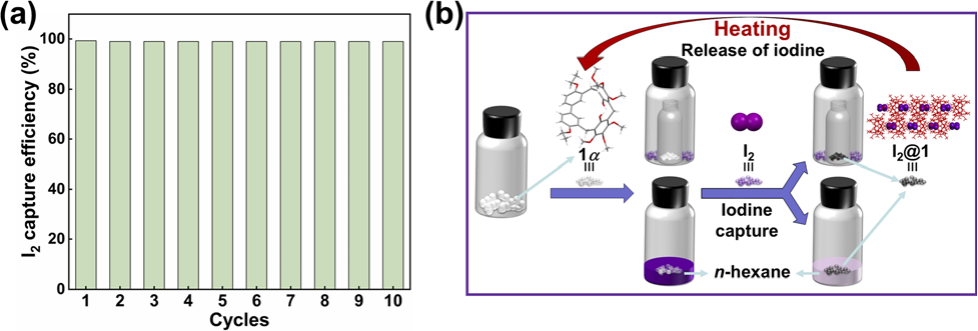
(a) The reusability of 1α in iodine capture. (b) Schematic diagrams represent the capture of iodine using 1α and the subsequent release of iodine.

Recyclability is a pivotal metric for evaluating the performance of adsorbents.^60,61^ For practical applications, the iodine adsorbent must perform well over multiple cycles without degradation. The iodine molecule was released by heating I_2_@1 at 125 °C. As indicated in ^1^H NMR, PXRD and FT-IR (Fig. S17–S19), the structure of I_2_@1 was restored to the original 1α after iodine release. Satisfactorily, the newly formed 1α could continue to be reused for capturing iodine and could be recycled at least 10 times without significant loss of performance (Fig. 5a). The recycling experiments indicated that 1α exhibited exceptional reusability across multiple cycles, thereby reinforcing its potential as an efficient iodine adsorbent in practical applications (Fig. 5b).

## Conclusions

In conclusion, we synthesized a novel hybrid[3]arene 1. The NACs derived from 1 (1α) exhibited reversible iodine capture behaviour. It was found that 1α enabled capture capacity for both iodine vapor and iodine in *n*-hexane, with maximum capture capacities of 1.87 g g^−1^ and 0.74 g g^−1^, respectively. The single crystal structure and electrostatic potential analysis suggested that the capture ability mainly came from C–H⋯I and O–H⋯I interactions between 1 molecule and iodine molecule. In addition, during the adsorption process, the morphology of 1α tended to form a rod-like stacked multilayer structure, which was favourable for iodine uptake by 1α. Furthermore, the reversible transitions between guest-free and guest-loaded structures improved the recyclability of 1α, which retained its capture efficiency over 10 cycles without noticeable loss in performance. Given the uncomplicated synthesis, favourable adsorption behaviour, and good recyclability of 1α, this system enriches the study of hybrid[3]arene-based NACs for iodine capture. It is expected that this work will provide a general strategy for constructing hybridarene-based NACs as efficient adsorbents for radioactive iodine adsorption, and be applied to related environmental remediation.

## Author contributions

R. Z. and J. Z. designed the experiments. R. Z. performed most experiments. R. Z. and W. L. analysed data. R. Z. and J. Z. wrote the manuscript. All authors agreed with the results and discussions presented in the manuscript.

## Conflicts of interest

The authors declare no conflicts of interest.

## Supplementary Material

SC-017-D6SC00236F-s001

SC-017-D6SC00236F-s002

## Data Availability

The data supporting this article have been included as part of the supplementary information (SI). Supplementary information: experimental procedures and characterization data. See DOI: https://doi.org/10.1039/d6sc00236f. CCDC 2296036 and 2493736 contain the supplementary crystallographic data for this paper.^62*a*,*b*^
