# Supplementary material for: Reversible iodine capture by nonporous adaptive hybrid[3]arene crystals
Source: Chem Sci. 2026 Apr 14;17(21):10653–7. doi: 10.1039/d6sc00236f (PMC13093743; doi:10.1039/d6sc00236f)
Supplement: SC-017-D6SC00236F-s001 [file SC-017-D6SC00236F-s001.pdf]

Supplementary Information (SI) for Chemical Science.
This journal is © The Royal Society of Chemistry 2026

Reversible iodine capture by nonporous adaptive hybrid[3]arene crystals

Ruike Zhang, Wenqian Liu and Jiong Zhou*

Department of Chemistry, College of Sciences, Northeastern University, Shenyang 110819, P. R. China

E-mail: zhoujiong@mail.neu.edu.cn

Supporting Information (17 pages)

1. *Materials* S2
2. *Methods* S2
3. *Synthesis of Hybrid[3]arene* ***1*** S4
4. *Crystallographic Data* S5
5. *Characterization of Activated* ***1*** ***(1α)*** S7
6. *Characterization of Iodine Release from* ***I_2@1*** S14
7. *References* S17

1. Materials

All reagents and solvents used in this work were obtained from commercial sources and were used without further purification. Activated crystalline hybrid[3]arene (**1**) was referred to as **1***α*. It was prepared by activating at 150 °C under vacuum for 4 h.

2. Methods

2.1. Solution NMR

^{1}H NMR and ^{13}C NMR spectra were recorded with Bruker Avance III HD 500 MHz or 600 MHz spectrometers with the deuterated solvent as the lock and the residual solvent or TMS as the internal reference. NMR spectra were reported as follows: chemical shift, multiplicity (s = singlet, d = doublet, t = triplet, q = quarter, m = multiplet, br = broad), coupling constant (J) and integration.

2.2. Powder X-Ray Diffraction

Powder X-ray diffraction (PXRD) data were collected on a Rigaku Ultimate-IV X-ray diffractometer operating at 40 kV/30 mA using the Cu Kα line (λ = 1.5418 Å). Data were measured over the range of 5−45° in 5°/min steps for a total of 8 min.

2.3. Thermogravimetric Analysis

Thermogravimetric analysis (TGA) was carried out using a Q5000IR analyzer (TA Instruments) with an automated vertical overhead thermobalance. The samples were heated at 10 °C/min using N_2 as the protective gas.

2.4. Single Crystal Growth

Single crystal of **1** was obtained as follows: 2 mg of **1** powder was dissolved in 2 mL of chloroform in an open 5 mL vial, which was placed in a sealed 20 mL vial containing 5 mL of *n*-hexane. Colorless single crystal was obtained in 1 day.

Single crystal of **I_2@1** was grown by slow evaporation: 2 mg of **1***α* powder was put in a small vial where 2 mL of chloroform solution of iodine (10 mg/mL) was added.

The resultant purple solution was allowed to free stand to give red crystal in 1 day.

2.5. Single Crystal X-ray Diffraction

Single crystal X-ray diffraction data were collected on a Bruker D8 VENTURE CMOS X-ray diffractometer with graphite monochromated Mo Kα radiation (λ = 0.71073 Å).

2.6. Gas Adsorption Measurement

Low-pressure gas adsorption measurements were performed on a Micromeritics Accelerated Surface Area and Porosimetry System (ASAP) 2020 surface area analyzer. Samples were degassed under dynamic vacuum for 12 h at 60 °C prior to each measurement. N_2 isotherms were measured using a liquid nitrogen bath (77 K).

2.7. High-Resolution Mass Spectrometry

High-resolution mass spectrometry (HR-MS) was performed on a Bruker 7-Tesla FT-ICR mass spectrometer (HR-MS, Bruker SolariX 7T).

2.8. Scanning Electron Microscopy and Energy-Dispersive X-ray Spectroscopy

The morphology of the samples was determined by scanning electron microscopy (SEM, JEOL 6500F). Determination of element distribution in samples by energy-dispersive x-ray spectroscopy (EDS, JEOL 6500F).

2.9. Ultraviolet-Visible Spectroscopy

Ultraviolet-visible (UV-Vis) absorbance spectra were recorded on a TU-1901 spectrophotometer (Beijing Purkinje General Instrument Co., Ltd., China).

2.10. Fourier Transform Infrared Spectroscopy

Fourier transform infrared (FT-IR) spectra were recorded on a PerkinElmer Spectrum One (B) spectrometer.

2.11. Melting Point Apparatus

The melting point was measured on an XT4 microscopic apparatus.

3. Synthesis of Hybrid[3]arene 1

Scheme S1. Synthetic Route to Hybrid[3]arene **1**.

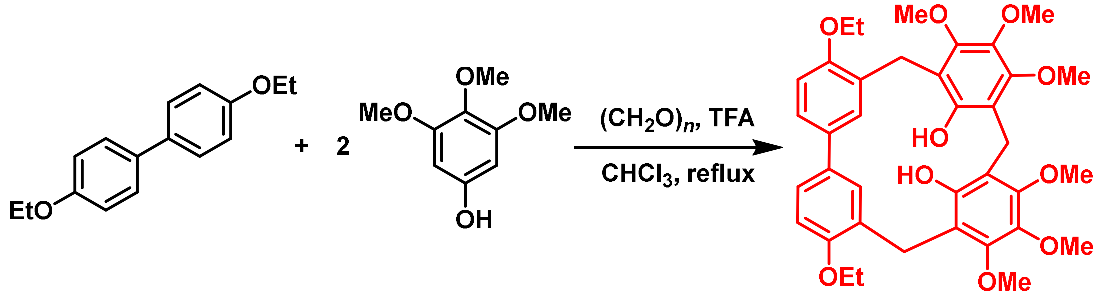

4,4'-biphenol diethyl ether   3,4,5-trimethoxyphenol   1

Synthesis of hybrid[3]arene **1**: To the solution of 4,4'-biphenol diethyl ether (2.42 g, 10.0 mmol) and 3,4,5-trimethoxyphenol (3.68 g, 20.0 mmol) in $CHCl_3$ (200 mL), paraformaldehyde (0.900 g, 30.0 mmol) and trifluoroacetic acid (TFA, 7.50 mL, 100 mmol) were added. The mixture was refluxed for 50 min, and the progress was monitored by thin-layer chromatography (TLC). The mixture was cooled to room temperature, and an excess of saturated $NaHCO_3$ solution was added to neutralize TFA. The organic phase was separated, and the crude product was purified by column chromatography (petroleum ether/ethyl acetate, *v*/*v* 5:1) to get **1** as a white solid (3.88 g, 60%), mp: 299.5-301.9 °C.

1 was dried under vacuum at 150 °C for 4 h to get activated crystalline **1** (**1**α). The ^{1}H NMR spectrum of **1**α was shown in Figure S1. ^{1}H NMR (500 MHz, chloroform-*d*, 293 K) δ (ppm): 7.20 (dd, J = 8, 2 Hz, 2H), 6.78 (s, 2H), 6.19 (s, 2H), 5.52 (s, 2H), 4.18 (d, J = 16 Hz, 2H), 4.14 (s, 2H), 4.08 (q, J = 7 Hz, 4H), 4.03 (s, 6H), 3.79 (s, 6H), 3.69 (d, J = 16 Hz, 2H), 3.56 (s, 6H), 1.46 (t, J = 7 Hz, 6H). The ^{13}C NMR spectrum of **1**α was shown in Figure S2. ^{13}C NMR (125 MHz, chloroform-*d*, 293 K) δ (ppm): 155.84, 151.63, 150.11, 149.05, 140.24, 132.94, 128.41, 127.17, 123.39, 116.73, 113.24, 110.55, 63.63, 61.81, 61.12, 61.08, 23.16, 18.08, 15.06. HR-MS was shown in Figure S3. *m*/*z* for $[M + Na]^+$ $C_{37}H_{42}NaO_{10}Na^+$, 669.2676; found 669.2667; error −2 ppm.

4. Crystallographic Data

Tab. S1. Experimental single crystal X-ray data for **1**.

Formula	**1**
Crystallization Solvent	chloroform
Collection Temperature (K)	273
Formula	$C_{37}H_{42}O_{10}$
Formula Weight	646.71
Crystal System	Orthorhombic
Space Group	Pbca
a [Å]	18.5767(3)
b [Å]	16.6595(3)
c [Å]	22.1764(3)
α [º]	90.00
β [º]	90.00
γ [º]	90.00
V [Å^3]	6863.12(19)
Z	8
D_{calcd} [g cm^{-3}]	1.252
Absorption coefficient (mm^{-1})	0.745
F(000)	2752
Theta range [º]	3.99-68.21
Reflections collected / unique	70843 / 6260 [R(int) = 0.0339]
Data / restraints / parameters	6260 / 0 / 424
Final R indices [I>2σ(I)]	R_1 = 0.1278, wR_2 = 0.1278
R indices (all data)	R_1 = 0.1341, wR_2 = 0.1341
Goodness-of-fit on F^2	1.004
Largest difference peak and hole [e.A^{-3}]	0.436 and -0.238
CCDC	2296036

Tab. S2. Experimental single crystal data for **I_2@1**.

Formula	**I_2@1**
Crystallization Solvent	chloroform
Collection Temperature (K)	273
Formula	$C_{37}H_{42}O_{10}I_2$
Formula Weight	900.51
Crystal System	Orthorhombic
Space Group	Pbca
a [Å]	20.5399
b [Å]	17.9690
c [Å]	24.9689
α [º]	90.00
β [º]	90.00
γ [º]	90.00
V [Å^3]	9215.6
Z	8
D_{calcd} [g cm^{-3}]	1.298
Absorption coefficient (mm^{-1})	11.094
F(000)	3600
Theta range [º]	3.54 - 65.08
Reflections collected / unique	68324 / 7733 [R(int) = 0.0620]
Data / restraints / parameters	7733 / 0 / 452
Final R indices [I>2σ(I)]	R_1 = 0.1998, wR_2 = 0.3749
R indices (all data)	R_1 = 0.2631, wR_2 = 0.3804
Goodness-of-fit on F^2	1.603
Largest difference peak and hole [e.A^{-3}]	4.730 and -2.666
CCDC	2493736

5. Characterization of Activated 1 Crystals (1*α*)

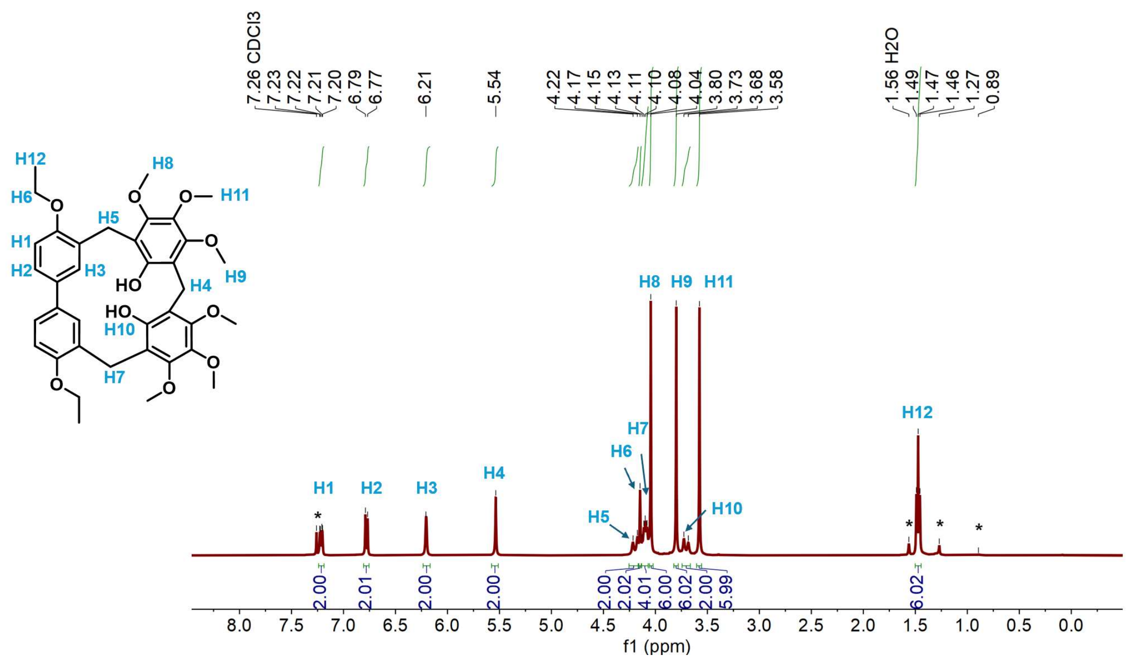

Figure S1. ^{1}H NMR spectrum (500 MHz, Chloroform-*d*, 293 K) of **1***α* (*: solvent).

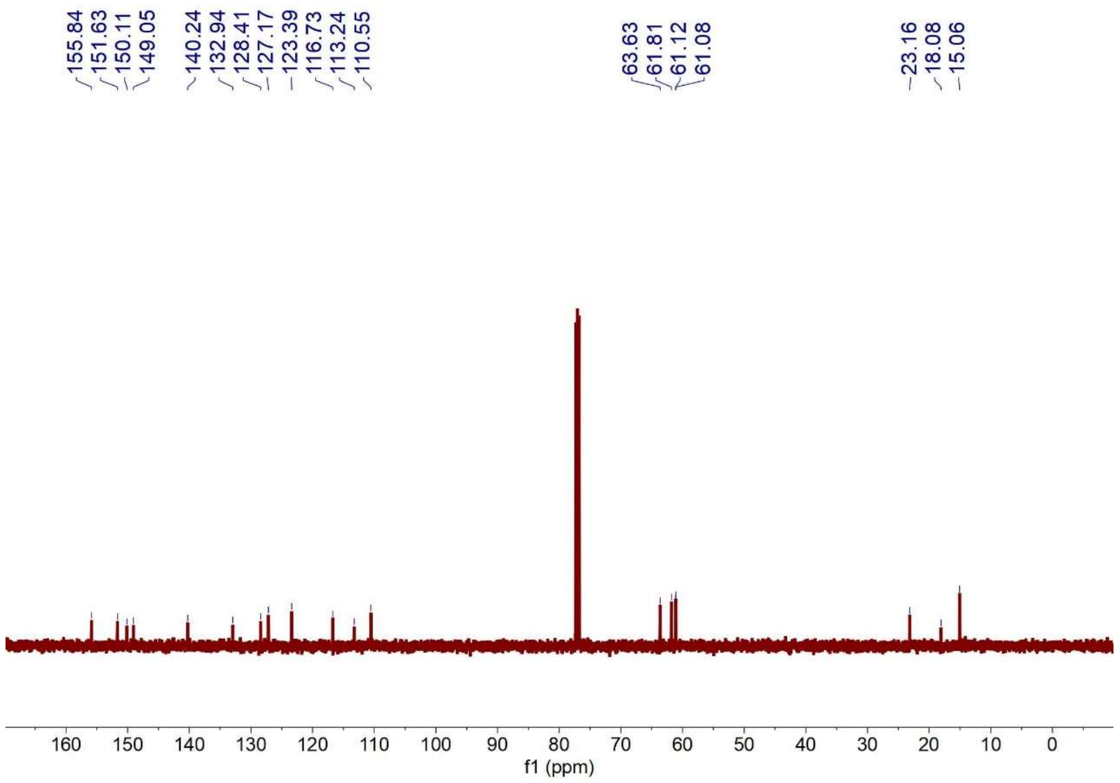

Figure S2. ^{13}C NMR spectrum (125 MHz, Chloroform-*d*, 293 K) of **1***α*.

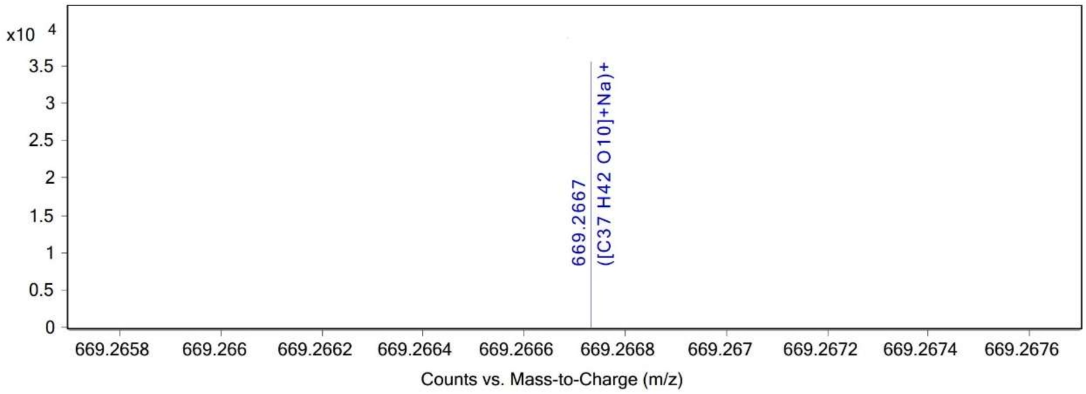

Figure S3. High-resolution mass spectrum of **1**α. Assignment of main peak: *m/z* for [M + Na]$^+$ $C_{37}H_{42}NaO_{10}Na^+$, 669.2676; found 669.2667; error −2 ppm.

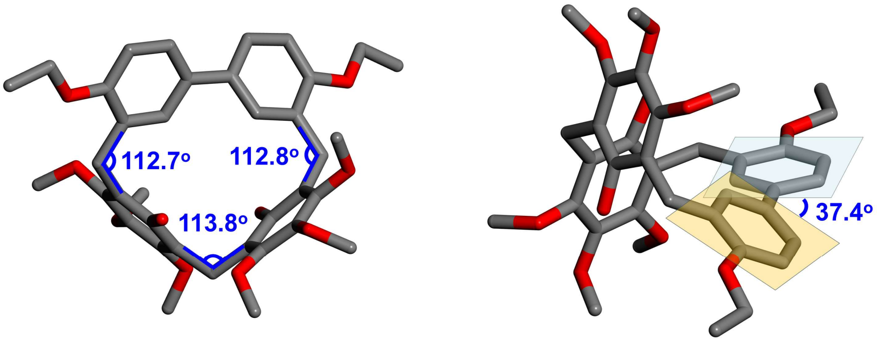

Figure S4. Data on bond angles and dihedral angles in **1**.

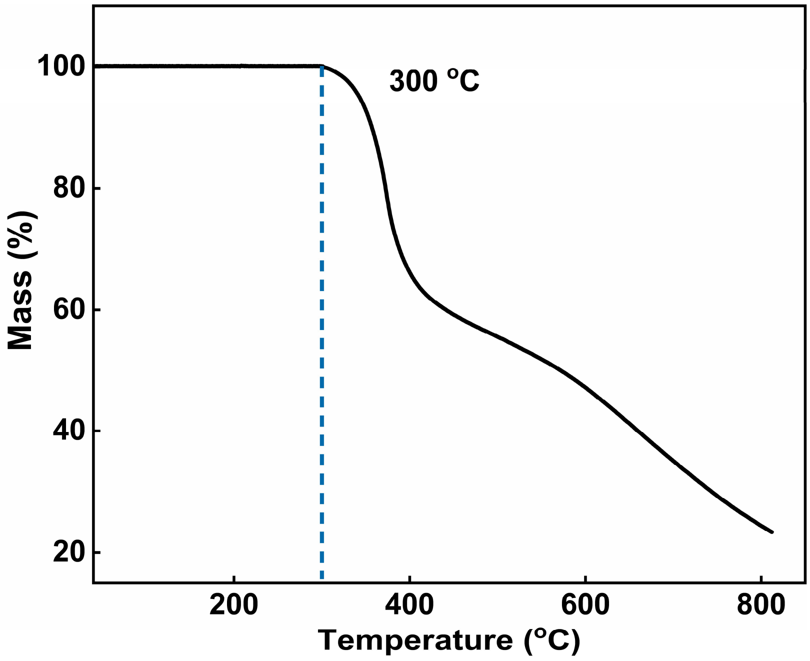

Figure S5. Thermogravimetric analysis of **H**α. The thermal decomposition of **1**α begins at 300 °C.

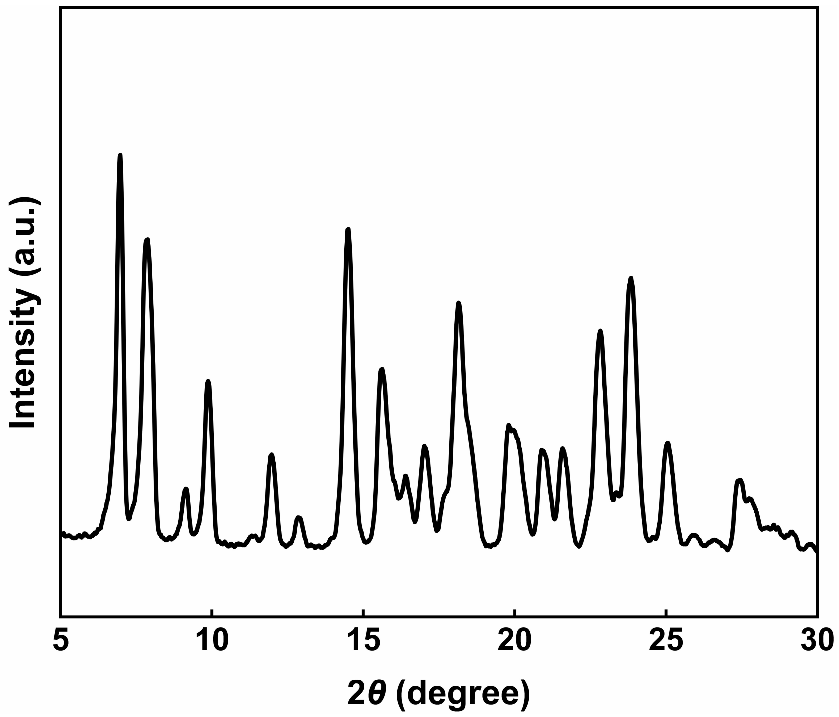

Figure S6. Powder X-ray diffraction pattern of **1**α.

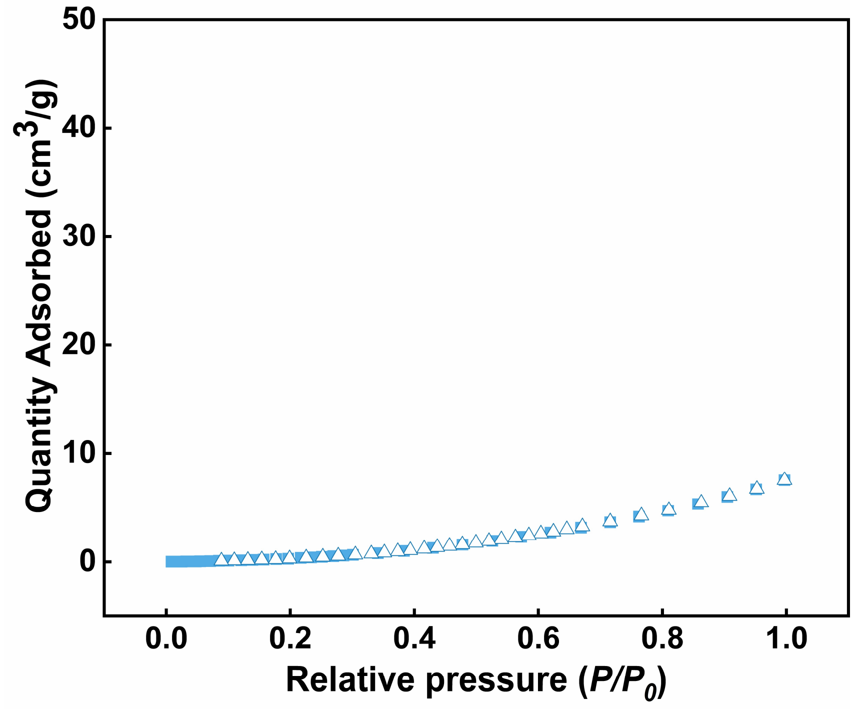

Figure S7. The N_2 adsorption-desorption curve of **1**α. **1**α shows a Brunauer-Emmett-Teller surface area of 1.03 m^2/g. The adsorption curve is represented by closed symbols and the desorption curve is represented by open symbols.

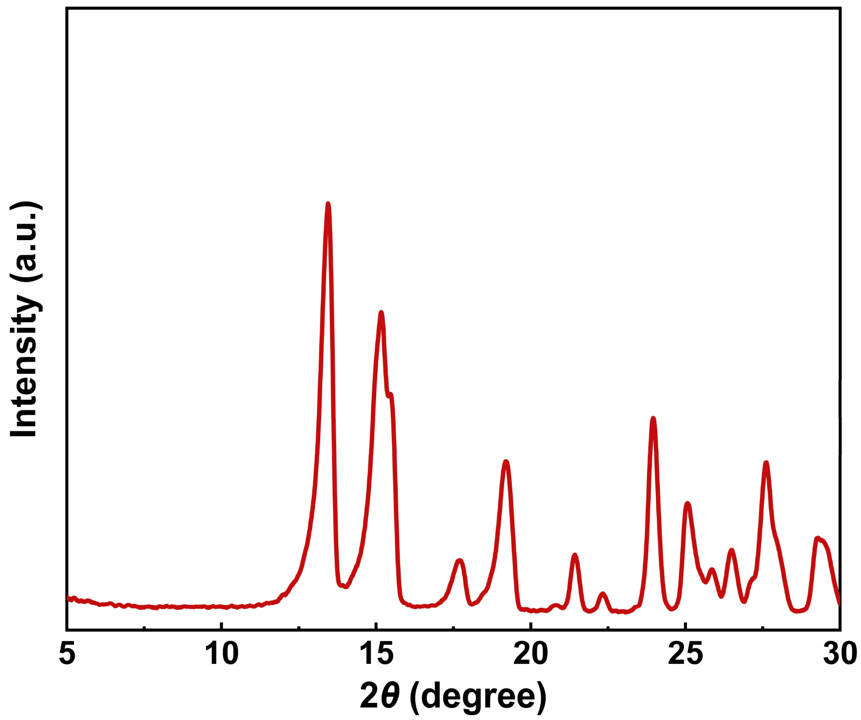

Figure S8. Powder X-ray diffraction pattern of $\mathbf{I_2}$@**1**.

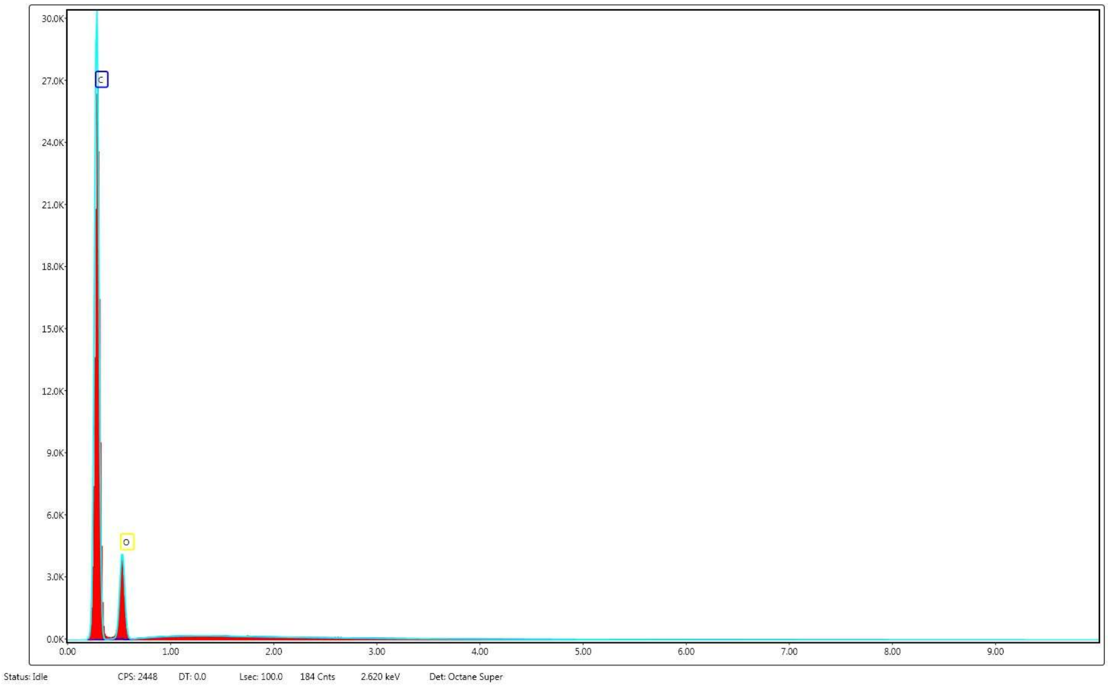

Figure S9. EDS analysis of **1**α.

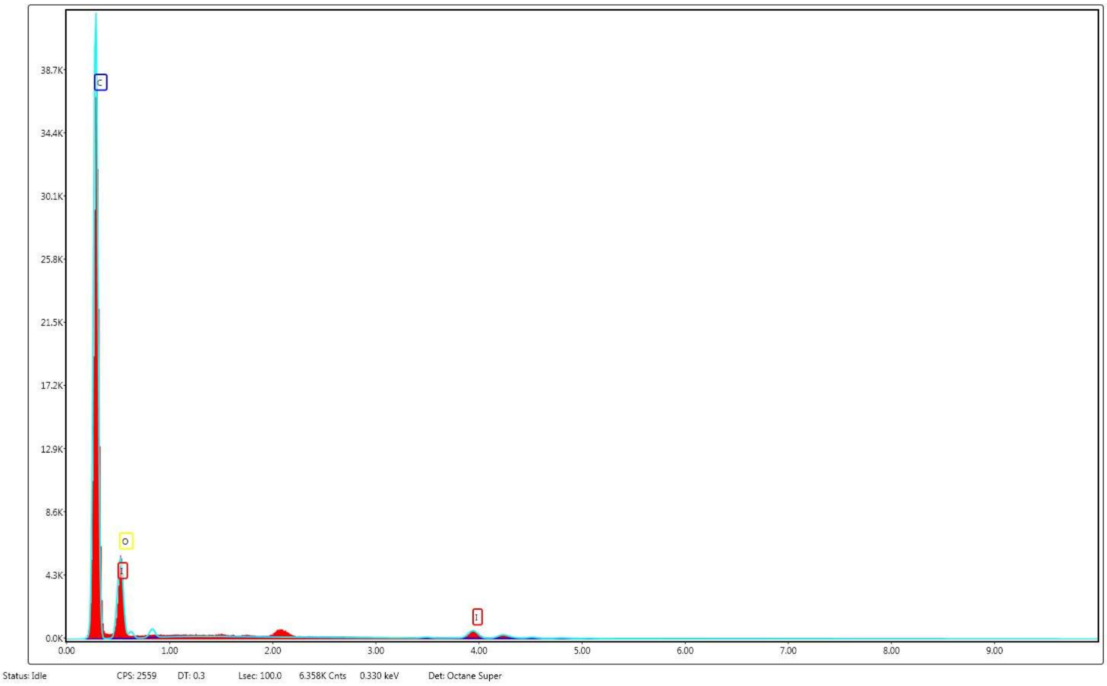

Figure S10. EDS analysis of **I_2@1**.

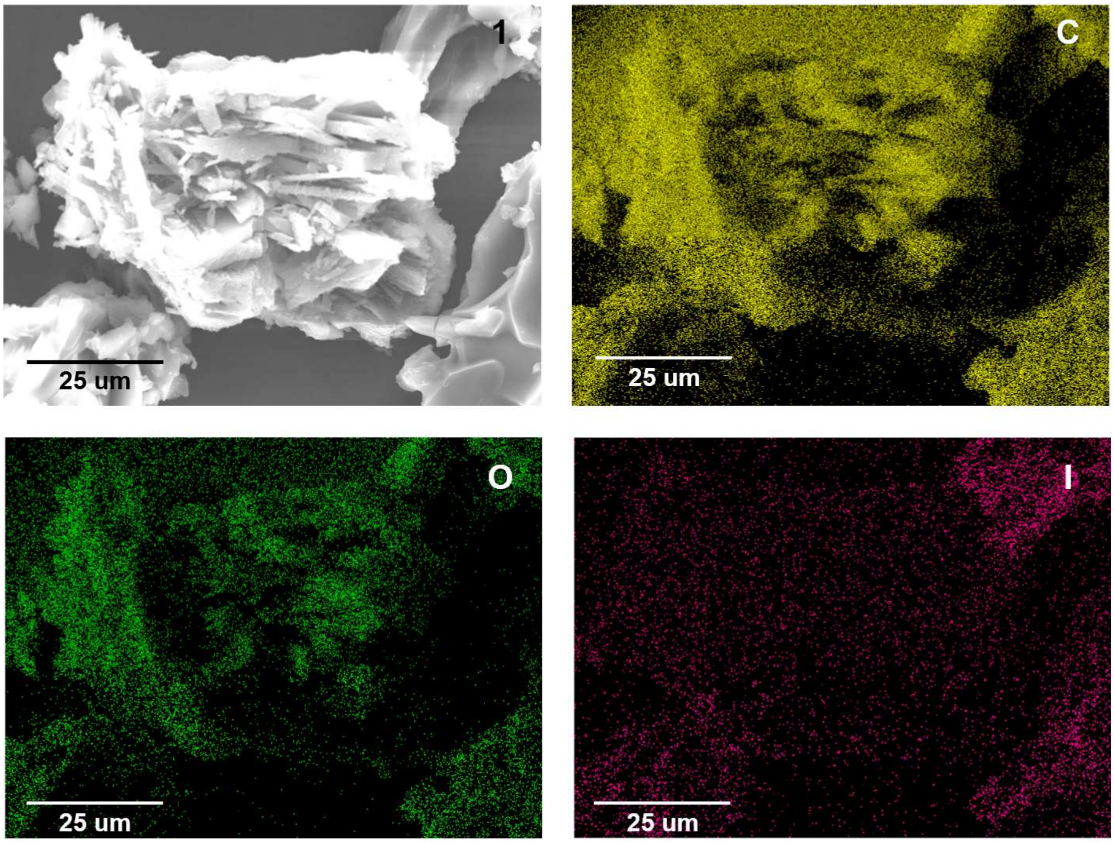

Figure S11. EDS mapping of **I_2@1**.

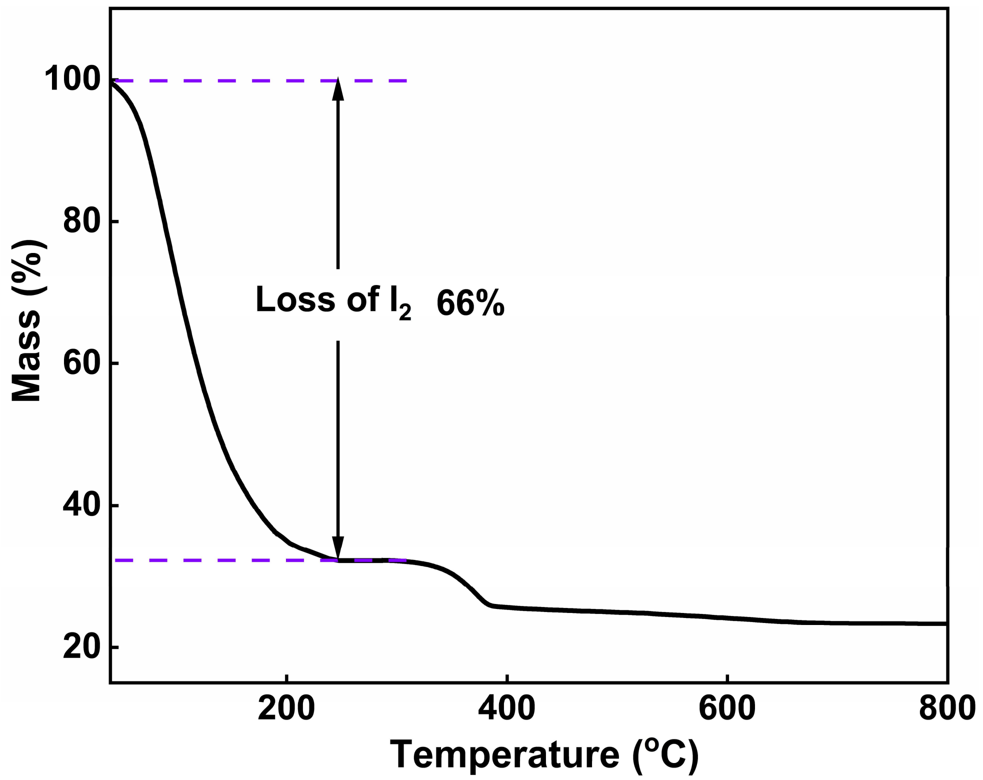

Figure S12. Thermogravimetric analysis of **I_2@1**.

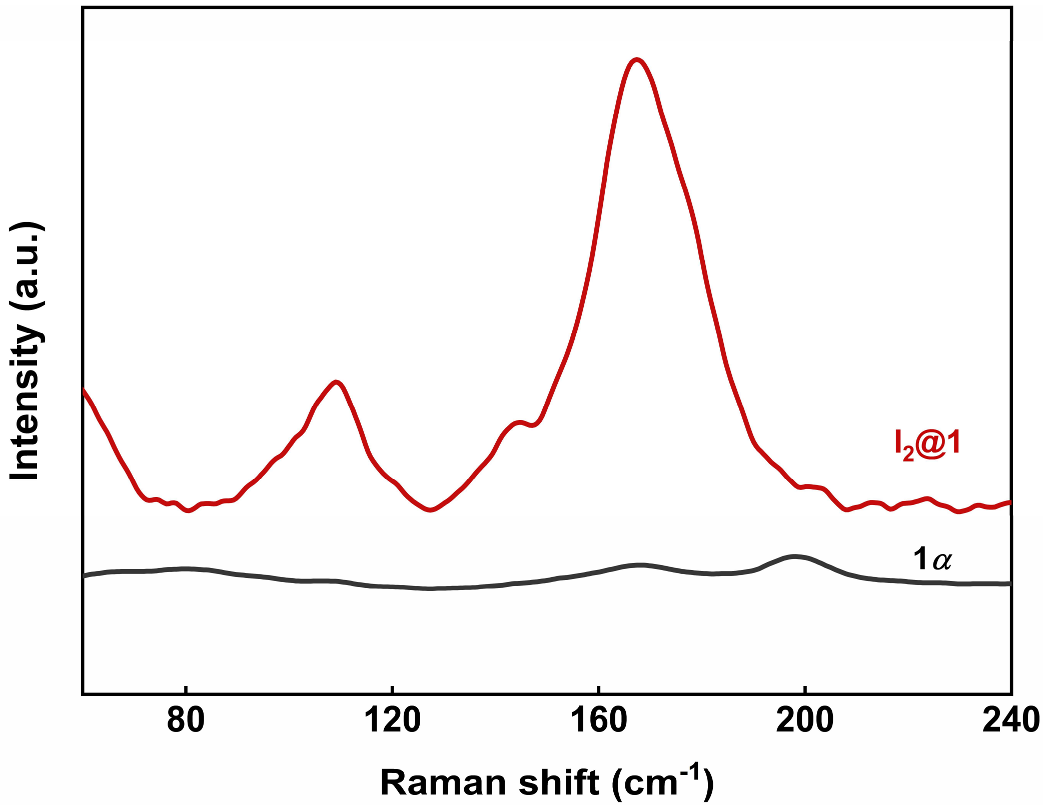

Figure S13. Raman spectra of **1**α and **I_2@1**.

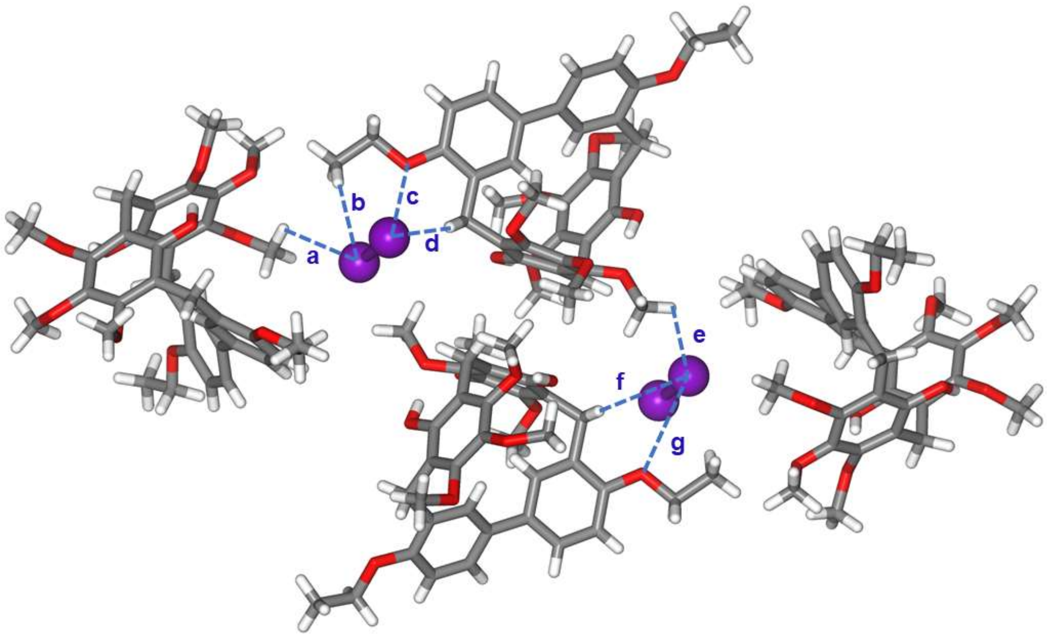

Figure S14. The host–guest complex formed by **1** and $\mathbf{I_2}$ is stabilized by C−H···I and O−H···I interactions. H–I distances: a = 3.479 Å, b = 2.841 Å, d = 3.137 Å, e = 2.373 Å, f = 3.295 Å. O−I distances: c = 3.756 Å, g = 3.646 Å.

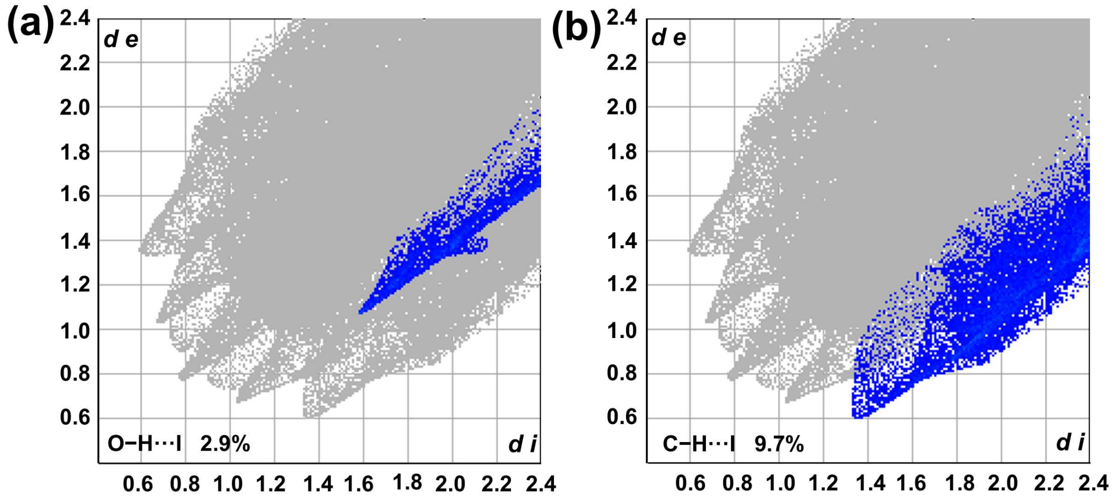

Figure S15. 2D fingerprint plots derived from Hirshfeld surface analysis of $\mathbf{I_2}$@**1**, showing the percentage contributions of: (a) O–H···I (host-to-guest) and (b) C–H···I (guest-to-host).

Table S3. Comparison of iodine adsorption capacities between hybrid[3]arene and other reported crystalline nonporous materials.

Materials	Temp. (°C)	Adsorption time (h)	Iodine uptake (g/g)	Ref.
Tetrahedral organic cage (**CC3**)	20	350	0.36	S1
Cobalt-bromo-naphthalene dicarboxylate (**Co-NDC-Br**)	75	24	0.452	S2
Cobalt-naphthalene dicarboxylate (**Co-NDC**)	75	24	0.346	
Phenyl-extended resorcin[4]arene (**OH-ExR4**)	25	0.5	0.625	S3
Tetracationic cyclophane (**BPy-Box^{4+}**)	75	24	3.99	S4
Tröger's base-benzophenone (**TBBP**)	80	12	3.43	S5
Per-ethylated pillar[6]arene (**EtP6**)	75	2	0.20	S6
Bisindole[3]arene (**MeBID[3]**)	75	24	5.12	S7
Per-ethylated aniline [2]arene (**EtAn[2]**)	75	30	7.35	S8
Nitrogen-bipyridine cage (**BPy-Cage**)	75	24	3.23	S9
Superphane cage (**SUPE–py–Imine–Cage**)	75	14	6.02	S10
Superphane cage (**SUPE–py–Amine–Cage**)	75	14	4.63	
Zn(II) coordination polymer (**Zn-IPDA**)	80	250	4.86	S11
Hybrid[3]arene (**1**)	77	0.7	1.87	This work

6. Iodine Release from I_2@1

Release of iodine from **I_2@1**: Two methods were employed to release the adsorbed iodine from **I_2@1**. In the solvent-release method, **I_2@1** was immersed in methanol for 2 h, leading to the desorption of iodine. In the thermal-release method, **I_2@1** was heated at 125 °C for 4 h to achieve iodine release. When the iodine release was completed, the

resulting solid was recycled and analyzed by ^{1}H NMR, PXRD and FT-IR spectroscopy.

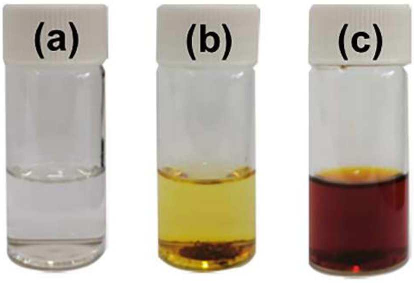

Figure S16. Photographs of color change when 3 mg of **I₂@1** solid was immersed in methanol. (a) Methanol; (b) initial release of iodine upon immersion in methanol; (c) iodine was released in methanol for 2 h.

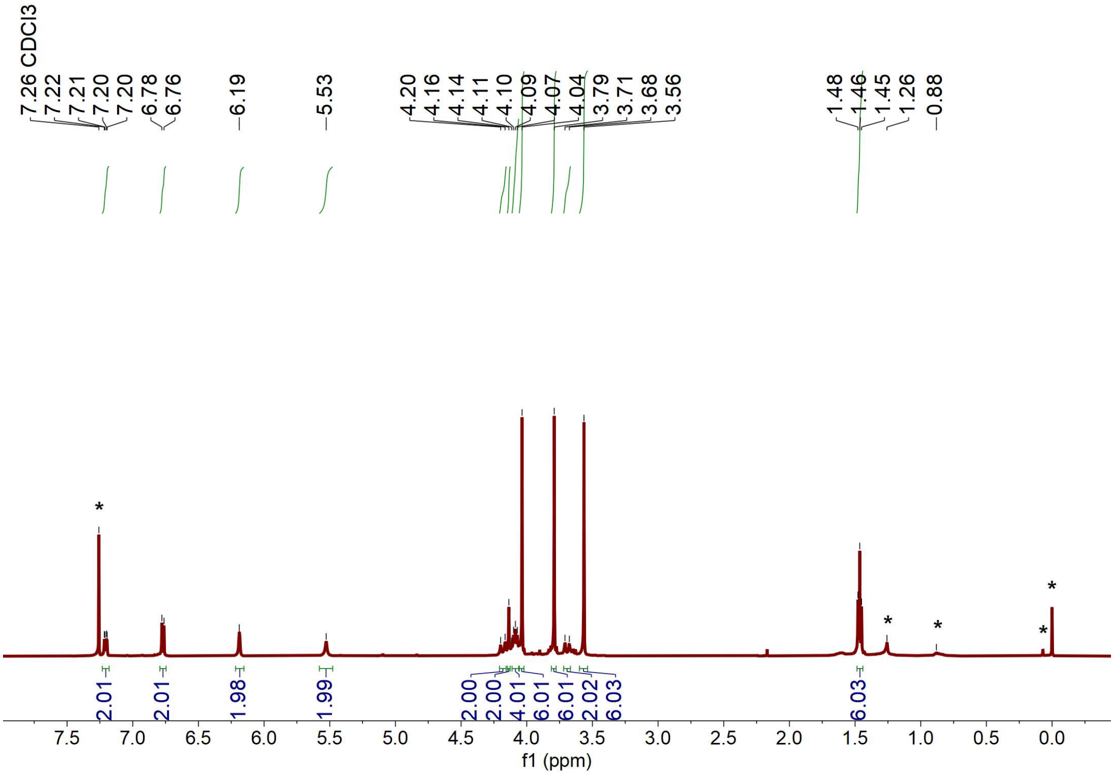

Figure S17. ^{1}H NMR spectrum (500 MHz, Chloroform-*d*, 293 K) of **I₂@1** after heating at 125 °C under vacuum for 4 h (*: solvent).

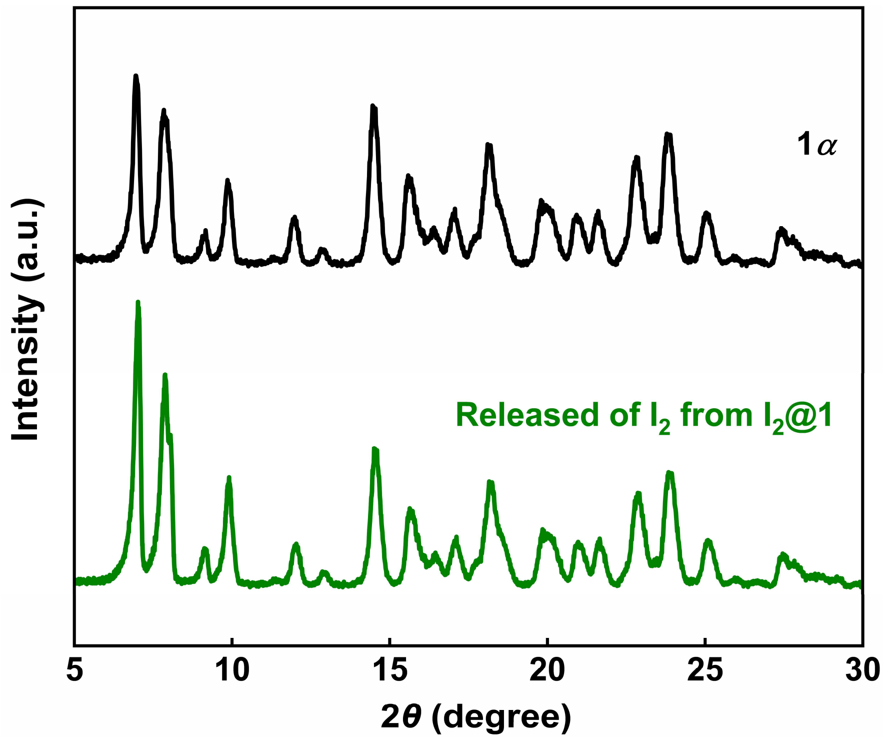

Figure S18. PXRD patterns of **1**α and **I$_2$@1** after releasing iodine.

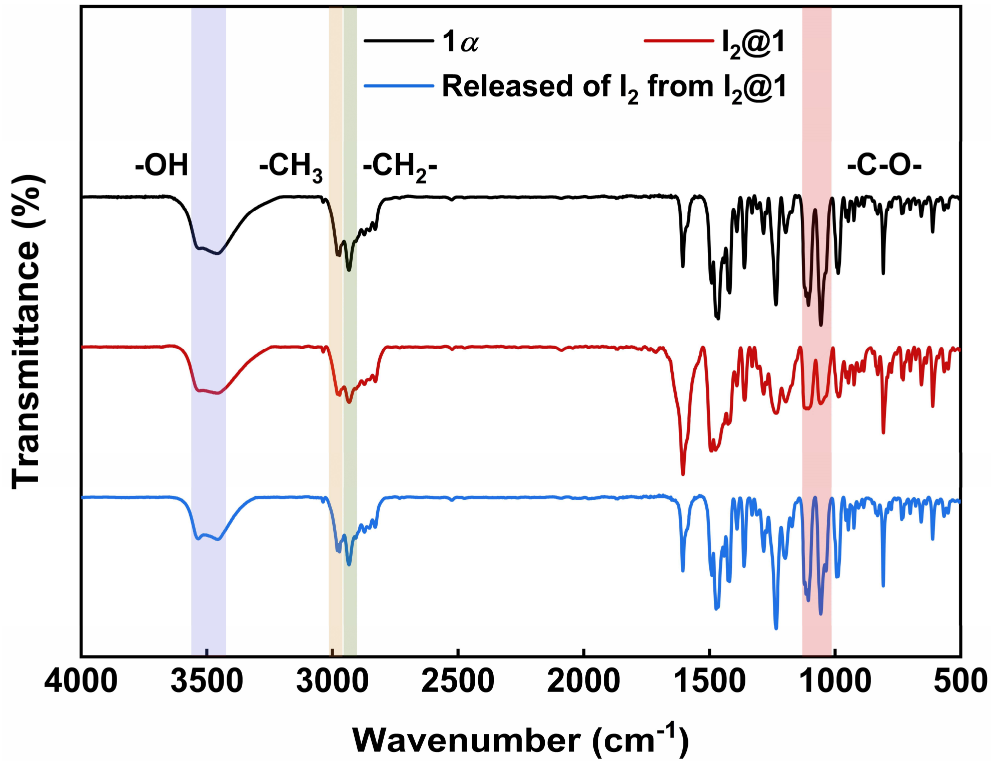

Figure S19. FT-IR spectra of **1**α, **I$_2$@1** and **I$_2$@1** after releasing iodine.

7. References

S1. T. Hasell, M. Schmidtmann and A. I. Cooper, *J. Am. Chem. Soc.*, 2011, **133**, 14920–14923.

S2. J.-Y. Liu, T.-P. Sheng, C. Li, Z. Wang, F.-R. Dai and Z.-N. Chen, *Cryst. Growth Des.*, 2022, **22**, 3182–3189.

S3. D. Li, G. Wu, Y.-K. Zhu and Y.-W. Yang, *Angew. Chem. Int. Ed.*, 2024, **63**, e202411261.

S4. B. Wu, Z.-W. Li, F. Lin, R. Tang, W. Zhang, H. Liu, G. Ouyang and Y. Tan, *J. Hazard. Mater.*, 2024, **465**, 133449.

S5. C. Shi, P. Niu, W. Xie, J. Jiao, Y. Li, R. Wang, C. Lin, L. Wang and J. Jiang, *Chem.–Eur. J.*, 2023, **29**, e202300410.

S6. K. Jie, Y. Zhou, E. Li, Z. Li, R. Zhao and F. Huang, *J. Am. Chem. Soc.*, 2017, **139**, 15320–15323.

S7. X. Yu, W. Wu, D. Zhou, D. Su, Z. Zhong and C. Yang, *CCS Chem.*, 2021, **4**, 1806-1814.

S8. P. Jin, W. Liang, Y. Rong, W. Wu, M. Gou, Y. Tang and C. Yang, *J. Mater. Chem. A*, 2023, **11**, 11126–11132.

S9. D. Luo, Y. He, J. Tian, J. L. Sessler and X. Chi, *J. Am. Chem. Soc.*, 2022, **144**, 113–117.

S10. W. Zhou, A. Li, M. Zhou, Y. Xu, Y. Zhang and Q. He, *Nat. Commun.*, 2023, **14**, 5388.

S11. C.-H. Zhang, B.-X. Zhou, X. Lin, J.-X. Wu, L.-H. Wu, S. Cai, J. Fan, W.-G. Zhang, Y. Yan and S.-R. Zheng, *Inorg. Chem. Front.*, 2024, **11**, 769–778.
